# Recurrent Cerebral Infarctions in Primary Sjögren Syndrome: A Case Report and Literature Review

**DOI:** 10.3389/fneur.2018.00865

**Published:** 2018-10-16

**Authors:** Jia-Ai Li, Hong-Mei Meng, Zhi-Tao Cui, Xue Wang, Jing Miao

**Affiliations:** ^1^Department of Neurology, The First Hospital of Jilin University, Changchun, China; ^2^Department of Geriatrics, The First Hospital of Jilin University, Changchun, China

**Keywords:** primary Sjögren syndrome, cerebral infarctions, central nervous system, anti-Ro(SSA) antibodies, vasculitis

## Abstract

Recurrent cerebral infarctions are extremely rare in patients with primary Sjögren syndrome. We report a 66-year-old woman who was admitted to our hospital due to acute cerebral infarction with exacerbation of dysphagia and right-sided hemiplegia as the main symptoms. In the past 3 months, she had developed cerebral infarction twice, even though she had no risk factors for atherosclerosis. She was eventually diagnosed with primary Sjögren syndrome based on a long history of dryness of the eyes and mouth, positive anti-Ro(SSA) antibodies, and the findings of a labial salivary gland biopsy. The response to pulse methylprednisolone therapy after recurrent cerebral infarctions was poor. Thus we consider primary Sjögren syndrome patients with central nervous system involvement should be treated as soon as possible.

## Introduction

Sjögren syndrome is a chronic, systemic autoimmune disorder characterized by lymphocytic infiltration of the exocrine glands (1). The condition can occur as a primary disease or be secondary to another connective tissue disease. Sjögren syndrome mainly involves the salivary and lacrimal glands, but can also affect other exocrine glands, organs, and systems (2), including the peripheral and central nervous systems (3). Central nervous system involvement is a rare complication of primary Sjögren syndrome (4) that is manifested by a variety of symptoms such as migraine, seizures, dementia, psychiatric disturbances, and cognitive dysfunction (5, 6). Thus far, recurrent strokes as a complication of primary Sjögren syndrome have not been reported. Here, we report an extremely rare case of recurrent strokes associated with primary Sjögren syndrome in a female patient.

## Background

**Figure 1:**
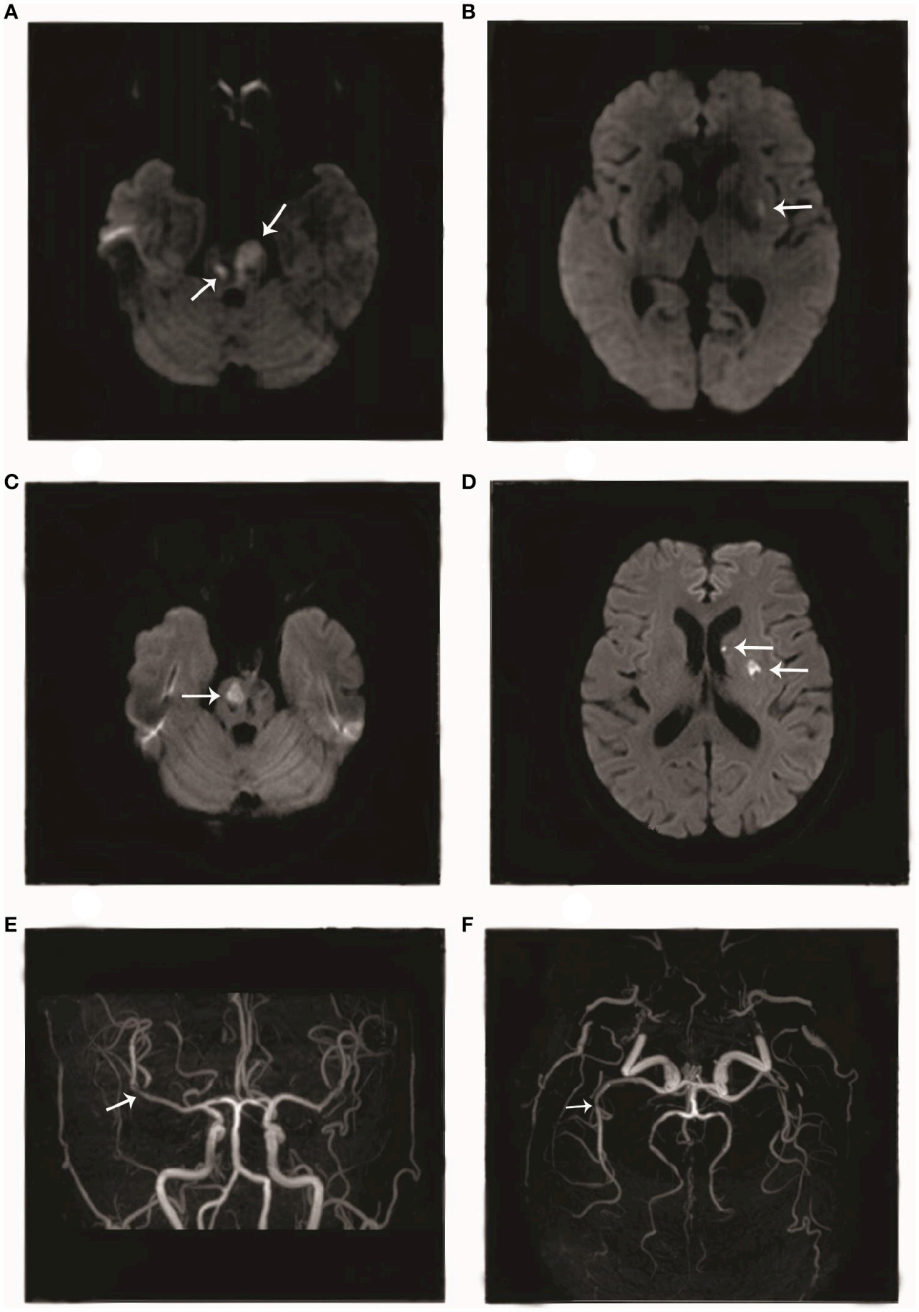
Imageological changes before admission. Diffusion-weighted imaging (DWI) performed in September 2017 showed hyperintense areas in the **(A)** right and left pons and **(B)** left insular cortex. DWI performed in November 2017 showed hyperintense areas in the **(C)** right pons and **(D)** left putamen. Magnetic resonance angiography performed in November 2017 showed **(E, F)** mild stenosis in the right middle cerebral artery.

**Figure 2:**
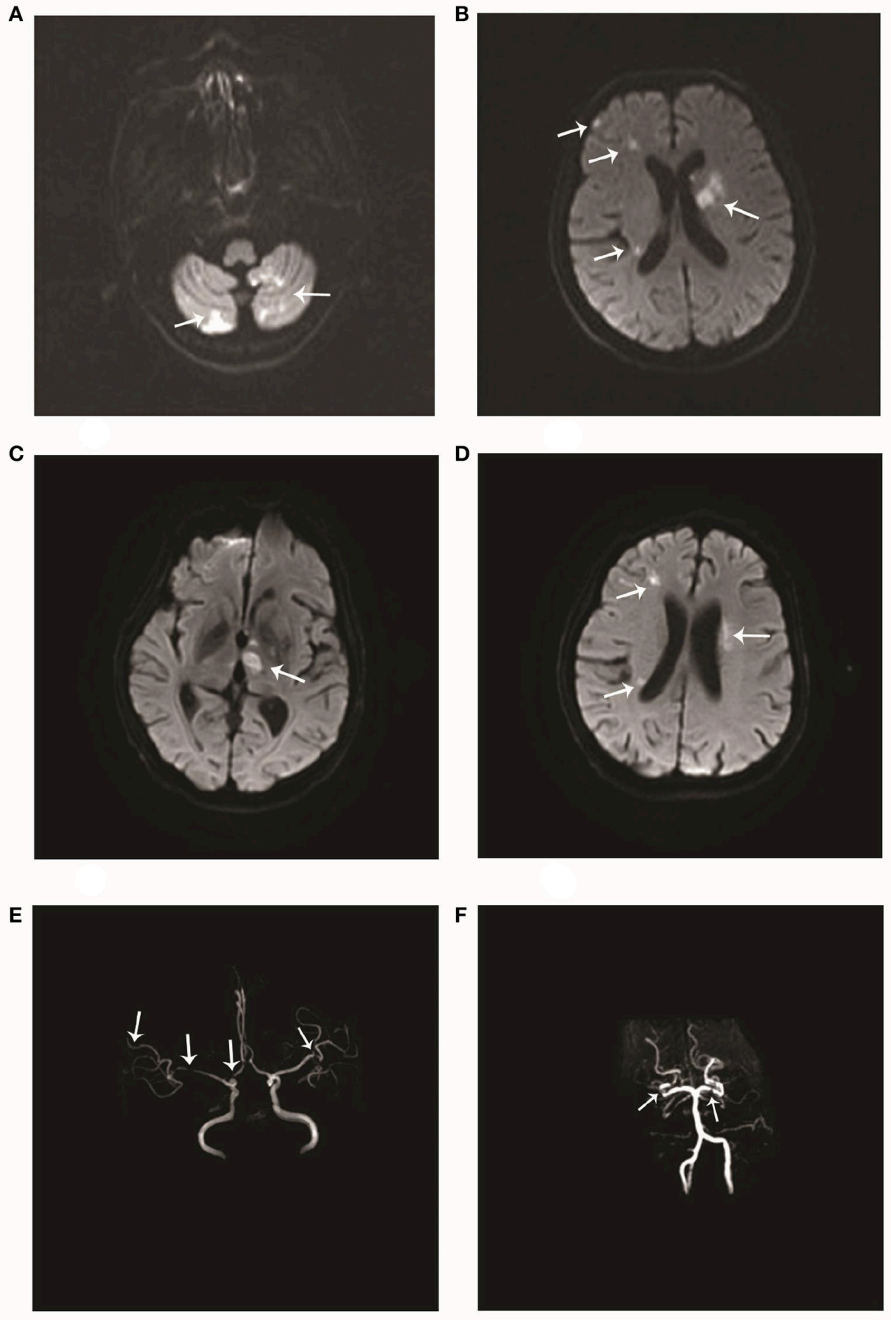
Imageological changes after admission. Diffusion-weighted imaging (DWI) performed after admission to our hospital in December 2017 showed multiple scattered hyperintense areas in the **(A)** right and left cerebellar hemispheres and **(B)** the left basal ganglia and right frontal and temporal lobes. Repeat DWI after 8 days showed multiple scattered hyperintense areas **(C, D)** near the lateral ventricles. Magnetic resonance angiography performed at the same time showed multiple stenoses in the **(E)** right anterior cerebral artery, right middle cerebral artery, and distal branch of the left middle cerebral artery, and **(F)** the right and left posterior cerebral arteries.

The patient was a 66-year-old woman. In September 2017, she had an episode of aphasia and right-sided hemiplegia, which was suggestive of a stroke. She therefore underwent magnetic resonance imaging (MRI) of the brain, which revealed a lacunar infarction in the right and left pons and the left insular white matter (Figures 1A,B). She was diagnosed with cerebral infarction and treated with butylphthalide, aspirin, and atorvastatin calcium for 2 weeks. After the treatment, she regained the ability to walk by herself and her speech improved. However, in November 2017, she again developed cerebral infarction, which manifested as dysphagia, and urinary and defecation disorders. She was hospitalized again, and as before, underwent routine treatment for cerebral infarction. A brain MRI after the second episode showed new infarct lesions in the right pons and the left putamen (Figures 1C,D). Furthermore, brain magnetic resonance angiography (MRA) showed a minor stenosis in the right middle cerebral artery, which did not explain the infarct sites (Figures 1E,F). Moreover, this time, the patient responded poorly to treatment with butylphthalide, aspirin, and atorvastatin. At 1 month after the second episode, she was brought to our neurology clinic due to exacerbation of dysphagia, right-sided hemiplegia, and altered mental status. On physical examination, she appeared lethargic, and disoriented to person, place, and time. She was unable to follow commands, and had right-sided gaze palsy and right-sided spastic hemiparesis. A brain MRI at our clinic showed more ischemic lesions in the right and left cerebellar hemispheres, pons, and frontal and temporal lobes, the left basal ganglia, and the right thalamus (Figures 2A,B).

**Figure 3:**
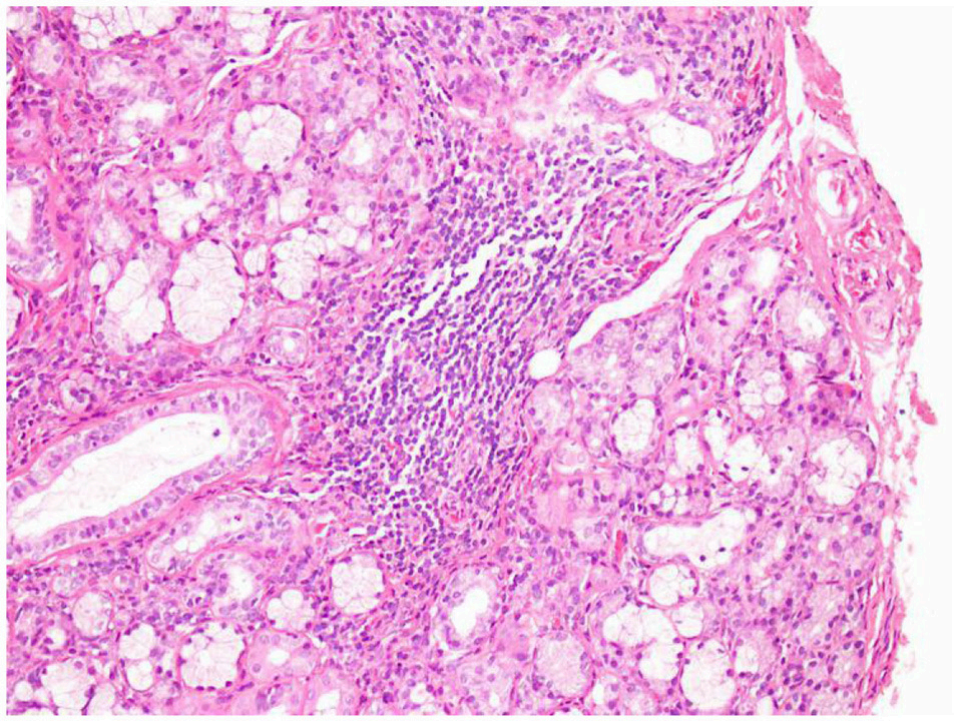
Minor labial salivary gland biopsy. Lymphocytic foci were found in the salivary gland tissue under 200 times microscope. The total area of the glands was 4 mm^2^ (hematoxylin and eosin).

The patient had no history of hypertension, diabetes, hyperlipidemia, coronary disease, or smoking or drinking. Significantly, the patient had dryness of the eyes and mouth since many years. On admission to our hospital, her blood pressure was 132/67 mmHg. A physical examination showed hemiplegia and hypertonia of the right limbs. The Babinski reflex was bilaterally positive. The remaining neurological examination could not be performed as the patient was not cooperative. In addition, there was edema of both lower limbs and bilateral pigmentation of the skin overlying the tibia. There were no obvious abnormities of the teeth. Serological examinations revealed positive anti-Ro(SSA) antibodies, and anti-nuclear antibodies at a titer of 1:3,200. The other laboratory results were as follows: anti-β2-glycoprotein antibodies, 152 RU/mL (normal range, 0–20 RU/mL); protein S activity, 52.9% (normal range, 60.0–130.0%); immunoglobulin G (IgG), 14.5 g/L (normal range, 7.0–17.00 g/L); and C-reactive protein, 29.50 mg/L (normal range, 0–3.5 mg/L). Thromboelastography revealed that the patient was sensitive to aspirin and clopidogrel. After obtaining written informed consent, we performed a lumbar puncture. The intracranial pressure was 125 mmH_2_O (normal range, 80–180 mmH_2_O). The cerebrospinal fluid study yielded the following findings: protein, 2.54 g/L (normal range, 0.15–0.45 g/L); IgG, 745 mg/L (normal range, 10–40 mg/L); and glucose, 2.1 mmol/L (normal range, 2.3–4.1 mmol/L). Oligoclonal bands were negative in both serum and cerebrospinal fluid. A brain MRA examination after admission to our hospital showed multiple stenoses in the right anterior cerebral artery, right middle cerebral artery, distal branch of the left middle cerebral artery, right and left posterior cerebral arteries, and basilar artery (Figures 2E,F), which is obviously different from the findings of the previous MRA. A repeat brain MRI after admission in our hospital (Figures 2C,D) revealed patchy foci of infarction in the right and left thalamus, cerebellar hemispheres, and pons, the right frontotemporal lobe, and the left basal ganglia. When compared to the previous MRI scans, the current examination clearly showed the progression of cerebral arterial stenosis. Considering the prolonged history of dry eyes and mouth combined with the examination findings, we highly suspected a diagnosis of primary Sjögren syndrome. Thus, after obtaining the consent of her family, we took a biopsy specimen from a minor labial salivary gland. Several lymphocytic foci were observed in the biopsy specimen (Figures 3). According to the 2012 American College of Rheumatology criteria 5 (7), the patient was diagnosed with primary Sjögren syndrome.

During hospitalization, the patient did not have symptoms of dyspnea; however, her D-dimer level was 5,773.0 μg/L (normal range, 0–232 μg/L), with a PaO_2_ of 69 mmHg (normal range, 83–108 mmHg) and a PaCO_2_ of 34 mmHg (normal range, 35–48 mmHg). Pulmonary computed tomographic angiography revealed emboli in the main pulmonary artery and a small branch of the right pulmonary artery, which was consistent with a diagnosis of pulmonary embolism. Additionally, multiple venous thromboses were found in both her lower extremities on ultrasonography.

Pulse methylprednisolone therapy was administered after the diagnosis of primary Sjögren syndrome with central nervous system involvement was established. Methylprednisolone was initially administered at a dose of 1,000 mg/day for 3 days, and then, the dose was reduced by half every 3 days. She was discharged on oral maintenance therapy with prednisolone acetate 80 mg/day. During hospitalization, she was also treated with cyclophosphamide 100 mg/day. Due to the pulmonary embolism and venous thrombosis in the lower limbs, she was treated with low-dose heparin for 20 days during hospitalization, followed by warfarin after discharge. After 1 month, the patient's clinical symptoms were not better, but the anti-Ro(SSA) antibodies and anti-β2-glycoprotein antibodies were negative, and the imaging showed no significant progress. So to some extent, the effect of hormone immunotherapy was not significant but immunotherapy also prevented the progression of the disease.

## Discussion

Sjögren syndrome is a systemic autoimmune disease characterized by keratoconjunctivitis sicca (dry eyes) and xerostomia (dry mouth) (8). This disease predominantly affects middle-aged women but can also occur in children, men, and the elderly (9, 10). When primary Sjögren syndrome involves the nervous system, it results in a wide spectrum of neurological manifestations. Central nervous system involvement in Sjögren syndrome is manifested by multiple sclerosis-like symptoms, including acute and chronic myelopathies, as well as by more diverse entities, such as cognitive dysfunction, subacute aseptic meningitis, encephalopathy, psychiatric symptoms, chorea, and seizure (11). The prevalence of primary Sjögren syndrome with central nervous system involvement varies widely from 10 to 67.5% (6, 11), as currently, the prevalence rate of this condition cannot be reliably estimated. Upon reviewing the literature on Sjögren syndrome with central nervous system involvement from 1960 to 2014, we found nine cases of stroke (12–15) and two cases of transient ischemic attacks (16, 17). However, none of the above cases involved recurrent strokes. Therefore, we concluded that stroke is an uncommon manifestation of Sjögren syndrome, and recurrent strokes are an even rarer manifestation. Thus, it is meaningful to report this case of a female patient who experienced recurrent strokes associated with primary Sjögren syndrome.

In our literature review, we also found two case reports of patients who had primary Sjögren syndrome and brain tissue damage. Yang et al. reported the case of a female patient with a 2-year history of primary Sjögren syndrome; she underwent systemic treatment after being diagnosed with the condition, but subsequently presented with sudden cerebral infarction in the left basal ganglia and parietal and occipital lobes (14). Niu et al. (18) reported the case of a 51-year-old woman who exhibited dizziness, slurred speech, and hemiplegia, and was eventually diagnosed with primary Sjögren syndrome. Her brain MRI revealed bilateral and symmetrical lesions in the basal ganglia, corona radiata, and corpus callosum. Neither patient had a history of hypertension, diabetes, hyperlipidemia, coronary heart disease, smoking, or drinking. Anti-Ro(SSA) antibodies were positive in both patients, and both patients showed an excellent response to hormone immunotherapy.

Like the above patients, our patient too tested positive for anti-Ro(SSA) antibodies. A strong correlation has been found between the presence of anti-Ro(SSA) antibodies and vasculitis in patients with primary Sjögren syndrome (15). Anti-Ro(SSA) antibodies are postulated to play a crucial role in the process of mediating or potentiating vascular injury in patients with Sjögren syndrome with central nervous system involvement (19). Alexander (20) suggested that inflammatory vasculopathy underlies the pathogenesis of this condition. Therefore, we think that anti-Ro(SSA) antibodies may mediate and potentiate inflammatory vasculopathy and eventually lead to severe vascular stenosis. The differences between the two MRA examinations (Figures 1E,F and 2E,F) in the present case report show the rapid progression of the disease in our patient.

The patients in the above two case reports did not have a history of venous or arterial embolism and did not test positive for anti-β2-glycoprotein antibodies. In contrast, our patient had a high level of anti-β2-glycoprotein antibodies and developed pulmonary embolism as well as multiple venous thromboses in both lower extremities. Anti-β2-glycoprotein antibodies often bind β2-glycoprotein I (β2GPI), which is a plasma protein that interacts with negatively charged phospholipids (21). Patients with anti-β2GPI antibodies may develop recurrent thromboembolism more easily than patients without these antibodies. Laplante et al. have proposed a potential mechanism for this finding. These authors think that antiphospholipid antibodies enhance tissue-factor expression by leukocytes and augment the adhesion of leukocytes to the arterial endothelium, thereby triggering embolic events (22). Therefore, we think that the recurrent cerebral infarctions in our patient are the result of a combination of vascular inflammatory injury and thrombosis. To some extent, it may be more difficult to treat primary Sjögren syndrome patients who have anti-β2-glycoprotein antibodies than those who do not.

Unlike the other two patients who only developed cerebral infarction once, our patient developed multiple infarctions and did not obtain any obvious benefit from pulse methylprednisolone therapy after the last cerebral infarction. This is consistent with the severe damage that the brain tissue sustains after frequent strokes. Thus, it is critical to identify the primary cause of the disease in stroke patients and intervene as soon as possible.

Usually, the symptomatic treatment of primary Sjögren syndrome is appropriate, and systemic treatment is reserved for patients with systemic manifestations. The treatment of primary Sjögren syndrome patients with central nervous system involvement is similar to that of patients with other systemic autoimmune diseases affecting the central nervous system and is based on experts' experience, mostly documented in case series. The treatment mainly includes immunosuppressive agents (high oral doses or intravenous pulses of steroids, cyclophosphamide, azathioprine, or mycophenolate mofetil) and possibly intravenous immunoglobulin and rituximab (23). According to our experience, as for the primary Sjögren syndrome patients with frequent strokes, the effect of hormone immunotherapy was not significant but can prevented the progression of the disease.

## Concluding remarks

We have described a case of primary Sjögren syndrome with recurrent cerebral infarctions. The patient did not respond to steroid treatment. The occurrence of recurrent cerebral infarctions seriously impacts the quality of life of the patient. Thus, primary Sjögren syndrome should be considered in the differential diagnosis of all patients who have inexplicable cerebral infarction, and treatment should be given to such patients as soon as possible.

## Ethics statement

This patient provided written informed consent agreeing to undergo treatment and allow the publication of the information that was described in the case report.

## Author contributions

The corresponding author is JM, who dominated this article. The first author is J-AL, who started and finished in writing and basic ideas. The second author is H-MM, who guided the completion of this article. The third author is Z-TC who provided a lot of guidance and corrections. The fourth author is XW who had provided lots of help in the process of improving the article.

### Conflict of interest statement

The authors declare that the research was conducted in the absence of any commercial or financial relationships that could be construed as a potential conflict of interest.
